# Unraveling the effects of gas species and surface wettability on the morphology of interfacial nanobubbles†

**DOI:** 10.1039/d2na00009a

**Published:** 2022-05-24

**Authors:** Kadi Hu, Liang Luo, Xiaoming Sun, Hui Li

**Affiliations:** Beijing Advanced Innovation Center for Soft Matter Science and Engineering, Beijing University of Chemistry Technology Beijing 100029 PR China hli@mail.buct.edu.cn; State Key Laboratory of Chemical Resource Engineering, Beijing University of Chemical Technology Beijing 100029 PR China

## Abstract

The morphology of interfacial nanobubbles (INBs) is a crucial but controversial topic in nanobubble research. We carried out atomistic molecular dynamics (MD) simulations to comprehensively study the morphology of INBs controlled by several determinant factors, including gas species, surface wettability, and bubble size. The simulations show that H_2_, O_2_ and N_2_ can all form stable INBs, with the contact angles (CAs, on the liquid side) following the order CA(H_2_) < CA(N_2_) < CA(O_2_), while CO_2_ prefers to form a gas film (pancake) structure on the substrate. The CA of INBs demonstrates a linear relation with the strength of interfacial interaction; however, a limited bubble CA of ∼25° is found on superhydrophilic surfaces. The high gas density and high internal pressure of the INBs are further confirmed, accompanied by strong interfacial gas enrichment (IGE) behavior. The morphology study of differently sized INBs shows that the internal density of the gas is drastically decreased with the bubble size at the initial stage of bubble nucleation, while the CA remains almost constant. Based on the simulation results, a modified Young's equation is presented for describing the extraordinary morphology of INBs.

## Abstract 2

Molecular dynamics simulations are performed to unravel the morphology dependence of interfacial nanobubbles on surface wettability and gas species.

## Graphical abstract

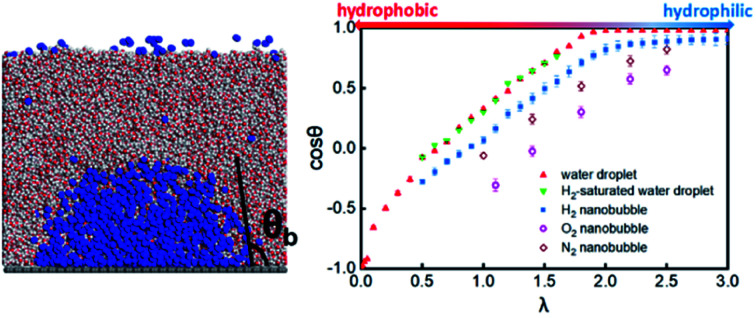

## Introduction

Interfacial nanobubbles (INBs), widely existing at solid–liquid interfaces, are considered a main factor to produce the hydrophobic interaction, playing key roles in determining various interfacial properties and causing many interfacial problems that need to be reconsidered.^1^ Therefore, INBs hold great potential in a wide range of industrial applications,^2^ such as cleaning and decontamination,^3^ mineral flotation,^4–6^ slip drag reduction,^7^ nanomaterial engineering,^8,9^ nanofluidics,^10,11^ biosensors,^12^ as well as energy conversion.^13^ Among their numerous unusual physicochemical properties, the morphology of INBs is of particular interest, due to the large inconsistency between the observed contact angle (CA, *θ*) of nanobubbles and macrobubbles. In electrochemical gas evolution reactions (GERs), it was found that the extraordinary morphology of the produced INBs can block the contact between electrolytes and electrode surfaces, severely affecting the mass transfer and conductance during the reactions.^14,15^ Recent studies have further shown the CA of INBs is also relevant to the overpotential in GERs.^16^ Thus, understanding the morphology of INBs is not only of great fundamental interest but also crucial importance in material applications and electrolysis.^17–25^

There have been great discrepancies in the observed behaviors of INBs under different experimental conditions. In most previous observations, INBs were found to have large and retarded CAs (on the liquid side).^26–31^ Atomic force microscopic (AFM) observations showed that the CA of INBs seems to have little relevance to the interfacial hydrophobicity but generally ranges from 150° to 170°, which is significantly larger than the corresponding CA of a macroscopic bubble on the same surface.^32–34^ It is believed that such oversized CA is a result of the joint actions of line tension, surface tension, and pinned contact line.^35–44^ The larger CA also gives the bubble a larger curvature radius, which can lower the internal Laplace pressure and help to stabilize the bubble.^42^ Theoretical models based on the surface pancakes and interfacial gas enrichment (IGE) were also proposed for understanding the anomalous CA of nanobubbles,^45–49^ which was further confirmed by the observed nanobubble-on-pancake object.^50^ On the other hand, some other experimental investigations have provided opposite views on the shape of INBs. For example, Wang *et al.* argued the nanobubbles should have a CA similar to a macrobubble, and the AFM characterization cannot reflect the real shape of the nanobubble due to the large curvature radius of the probe tip.^51^ Besides the tip effect of AFM, it was found that many other uncontrollable conditions, such as contamination, heterogeneity of surface, chemical composition of liquid, gas content in the bubble, and so on, may also lead to the contradictive experimental results,^29,52–56^ indicating there is a certain gap between the experimentally observed morphology of INBs and their real appearance.

In addition to the experimental investigations, atomistic molecular dynamics (MD) simulation is also a promising tool to investigate the dynamics of solid–liquid–gas interfaces at the molecular level, and it has been widely employed to study the behaviors of INBs in recent years.^15,57–60^ In the simulation of an argon vapor bubble on a solid surface, Maruyama *et al.* found the CA of INBs on solid surface could be modified by the potential parameters between gas and solid.^57^ Nagayama *et al.* revealed that the bubble nucleation also shows a remarkable dependence on the solid–liquid interfacial interaction.^58^ Zhang *et al.* explored the size dependence of the isothermal compressibility of the gas bubble, leading to a size-dependent bubble CA.^59^ Lohse *et al.* revealed that INBs are stabilized by a non-equilibrium mechanism, where the dense layer of gas at the solid–liquid interface effectively changes the substrate chemistry, leading to the universal CA.^60^ Although these simulations have already illustrated that the CA of nanobubbles is relevant to surface wettability and bubble size, systematic studies of the quantitative relationships between morphology of INBs and the possible determinant factors, including gas species, surface energy, and bubble size, are still lacking.

In the present work, we carry out a series of atomistic MD simulations to investigate the morphology of INBs formed from the most common types of gas molecules produced in GERs (*e.g.*, H_2_, O_2_, CO_2_, and N_2_) on flat solid surfaces. The quantitative relationships between nanobubble CA and gas species, bubble size, and surface energy are systematically established. The key structural parameters, including density distribution and internal pressure of INBs, are also observed at various CAs. The present simulation work has given a complete illustration of the morphology of INBs, which sheds important new light on relevant interfacial physical phenomena and applications.

## Methods

The large-scale atomic/molecular parallel simulator (LAMMPS) software was used for the MD simulations.^61^ There are two kinds of simulation systems in the present work: one is the interfacial nanobubble system, and the other is the water droplet system on the same substrate (Fig. S1, ESI†). The substrate (in the size of 14 × 14 nm^2^) is an atomic flat surface in a graphene-like honeycomb lattice. Periodic boundary conditions are employed in the *x* and *y* directions, and the mirror boundary condition is used in the *z* direction. In the nanobubble simulation, the space around the bubble is full of water molecules, and at the same time, a vacuum space is also added to the simulation box to create a liquid–vapor interface far from the bubble.

The water molecules are treated using the SPC/E model.^62^ The Lennard–Jones potential 
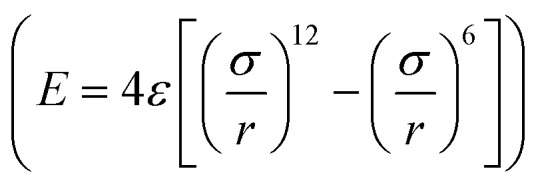
 with a cutoff of 12.0 Å is employed for the van der Waals interaction. The interaction parameters are shown in Table S1,† and parameters between different types of atoms are calculated through the Lorentz–Berthelot combining rules 
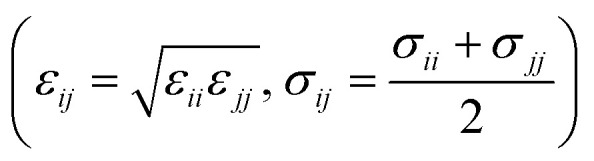
. The particle–particle particle-mesh (PPPM) solver is employed to compute the long-range coulombic interactions and the 1/*r*^6^ interactions.

All the MD simulations are performed in constant-volume and constant-temperature (NVT) ensembles. The Nosé–Hoover thermostat is employed to control the temperature at 300 K.^63^ A cubic cluster consisting of water molecules or gas molecules is initially placed with the shortest distance of ∼2.5 Å above the graphene substrate to relax to a droplet or droplet-like bubble. The substrate is fixed during the MD simulation. The time step is set as 1.0 fs. After full relaxation, simulations with time >20 ns are performed to make sure all these systems can reach the equilibrium states.

## Results and discussion

### Morphology of INBs with different gas species

**Fig. 1:**
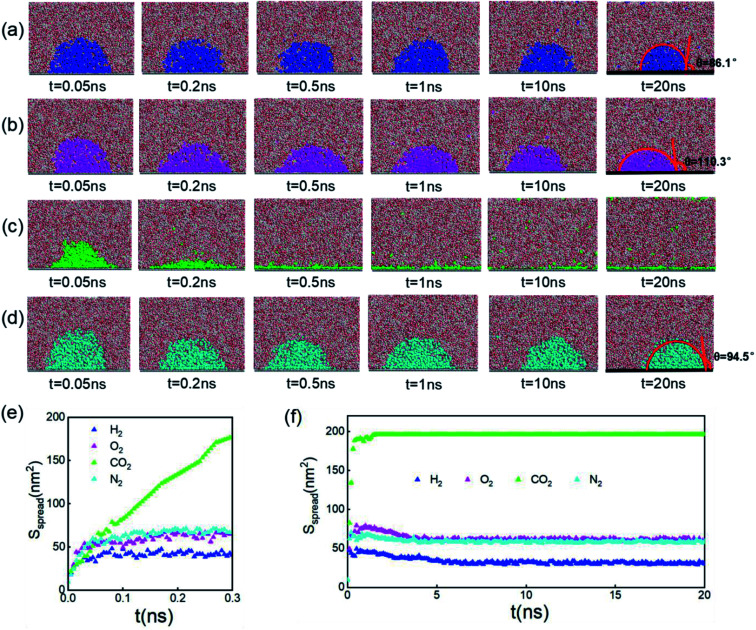
Time evolution of (a) H_2_-, (b) O_2_-, (c) CO_2_-, and (d) N_2_-INBs on the graphene surface starting from a cubic morphology. (e) The evolution of spreading areas of INBs with different gases during the first 0.3 ns and (f) the 0–20.0 ns simulation periods. (a)–(d) Denote the side view of the snapshots, while the plane view of the snapshots is shown in Fig. S2.†

Since inhomogeneity always exists on real surfaces, the surface pinning often plays a key role in the formation of INBs. The pinning positions can fix the triple-line of bubbles, thereby apparently affecting their morphology and CA, leading to the uncertainty of characterization of the pristine morphology of INBs. To probe the intrinsic behaviours of INBs without any wetting hysteresis, we employ a homogeneous atomic-flat model surface in the graphene lattice, whose surface energy can be adjusted by modifying the van der Waals parameters. Firstly, we compare the evolution of INBs filled with different types of gas (H_2_, O_2_, CO_2_, and N_2_) on the graphene surface. As shown by the MD trajectory in Fig. 1a, the initial cubic H_2_ cluster turns into a hemisphere in a short time (<0.05 ns) and remains almost unchanged from 0.5 to 20 ns, indicating the H_2_-INB is very stable with a constant CA (from the water side) close to the macrobubble on the substrate. Similarly, the O_2_- and N_2_-clusters (Fig. 1b and d) also show similar stability. By contrast, the CO_2_ cluster demonstrates a complete wetting behaviour (Fig. 1c), where the CO_2_ bubble continuously spreads on the substrate until it becomes a thin film formed by one or two layers of gas molecules. The different morphology evolutions of H_2_/O_2_/N_2_-INBs and CO_2_-INB are also reflected by the evolution of their contact areas on the substrate. As shown in Fig. 1d and e, the contact area of H_2_-INB reaches a maximum value within the first 1 ns, while the contact area of CO_2_-INB is increased until it covers the whole area of the simulation box rapidly. It is noteworthy that after the initial extension, the contact area of INBs slightly decreases in the next 5 ns, due to dissolving of gas molecules (H_2_, O_2_, and N_2_) in the bulk water before the solution is completely saturated, and after that, the radius remains a constant during the rest of the simulation period (5–20 ns).

**Fig. 2:**
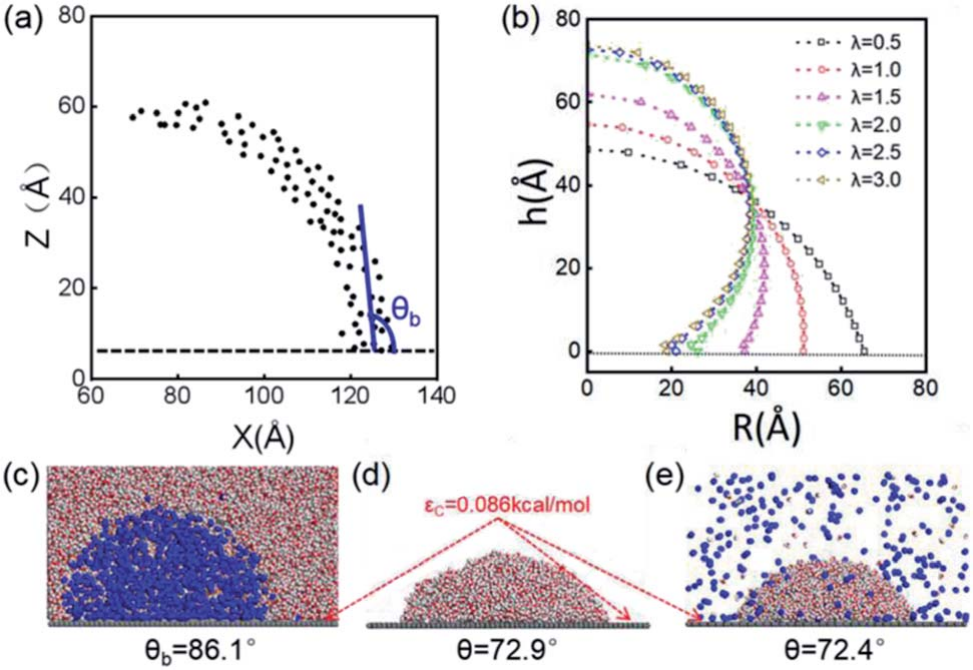
(a) Schematic presentation of contact angle calculation. Black points represent the half-water-density areas to characterize the surface of the nanobubble or water droplet; the red curve is a fit to black points; and the blue line denotes the tangent of the bubble/droplet surface. (b) Shapes of INBs on the surface with different wettabilities. *λ* is a coefficient of substrate wettability; we multiply the initial energy parameter of the substrate *ε*_C_ = 0.086 kcal mol^−1^ by *λ* (0.01–3.0) to change the surface wettability from hydrophobic to hydrophilic. The corresponding snapshots are shown in Fig. S3.† Snapshots of (c) H_2_ nanobubble, (d) water nanodroplet and (e) water nanodroplet with excessive H_2_ molecules. H_2_ molecules are represented by blue balls; H_2_O molecules consist of red (O) and white (H) balls, and graphene atoms are denoted by the gray balls.

The stable shapes during the last periods of simulations are used to statistically evaluate the CAs of H_2_-, O_2_-, and N_2_-INBs. The whole simulation box is split into cubic meshes with a lattice size of 3.0 Å, and the local water density in each mesh is recorded based on the MD trajectories. The CA is derived by fitting the water–air interface with the grids, with the local density being half of the bulk water, as shown in Fig. 2a.^64^ The obtained CAs for the three bubble species are in the order of CA(O_2_-INB) (*θ* = 110.3°) > CA(N_2_-INB) (*θ* = 94.5°) > CA(H_2_-INB) (*θ* = 86.1°), as shown in Fig. 1a–d, while the gas film of CO_2_ can be considered to possess a super large CA (*θ* ≈ 180°). Consistent with previous reports,^57^ the bubble CA value is strongly dependent on the strength of interaction between the substrate and adsorbed molecules, which is determined by the energy parameters (*ε*) of the Lennard–Jones (LJ) potential. As listed in Table S1,† the gas–solid interaction is stronger than the liquid–solid interaction in the CO_2_–H_2_O system, indicating the CO_2_–solid interface is more energetically favourable than the water–solid interface. In fact, the INB filled with CO_2_ may form a film structure with the triple line pinned by some surface pinning sites, thereby yielding a super large CA. On the contrary, the gas–solid interaction is weaker than the liquid–solid interaction in the H_2_–H_2_O, O_2_–H_2_O, and N_2_–H_2_O systems, indicating all three gas species can form stable INBs on an atomic flat surface. It is also found that the LJ potentials of H_2_, N_2_, and O_2_ follow the same order as the CA values of INBs with different gas species.

According to the present simulations, different species of gas molecules demonstrate significantly different behaviors in forming INBs: CO_2_ prefers to form a gas film rather than a stable nanobubble at the water–solid interface; O_2_ and N_2_ are willing to adopt INBs with larger CAs; and H_2_ forms an INB with a smaller CA. Such result is consistent with the previous model study of Molinero *et al.*,^15^ who found three stationary states of nanobubble nucleation: micropancakes (when gas–solid interaction is larger than liquid–solid interaction), interfacial nanobubbles, and solution nanobubbles (when gas–solid interaction is smaller than liquid–solid interaction).^21^ Furthermore, it is worth noting that more CO_2_ molecules, rather than O_2_ and N_2_, are found to be dispersed in the water solution, confirming the higher solubility of CO_2_ (Fig. 1b–d).

### INB morphology *vs.* surface wettability

The surface wettability of the solid substrate is another key factor determining the morphology of INBs. We compare the morphology of INBs filled with various gas species on surfaces with different surface energies. It is found that the CO_2_-INBs always form gas films on both hydrophobic and hydrophilic surfaces. By contrast, the H_2_/O_2_/N_2_-INBs display a macrodroplet-like shape, whose intrinsic CA can be modified by the strength of interfacial van der Waals interaction. Consistent with the method in previous works,^57,64,65^ a coefficient *λ* is employed to multiply the energy parameter *ε* of the substrate to tune the hydrophobicity of the surface model. When the value of *λ* is sufficiently large, the substrate becomes superhydrophilic, while a superhydrophobic substrate is obtained when *λ* approaches 0. As demonstrated in Fig. 2b, the statistic CA of INBs can be greatly varied by the value of *λ*. Although the pancake shape is not formed, as compared in Fig. 2c and d (*ε*_C_ = 0.086 kcal mol^−1^), the CA of H_2_-INB (*θ* = 86.1°) is apparently larger than the corresponding CA of pure water droplet (*θ* = 72.9°). Furthermore, the H_2_-saturated water droplet (Fig. 2e) shows an identical CA (*θ* = 72.4°) to the pure water droplet, excluding the possibility of the drop in water surface tension being caused by dissolved H_2_.

**Fig. 3:**
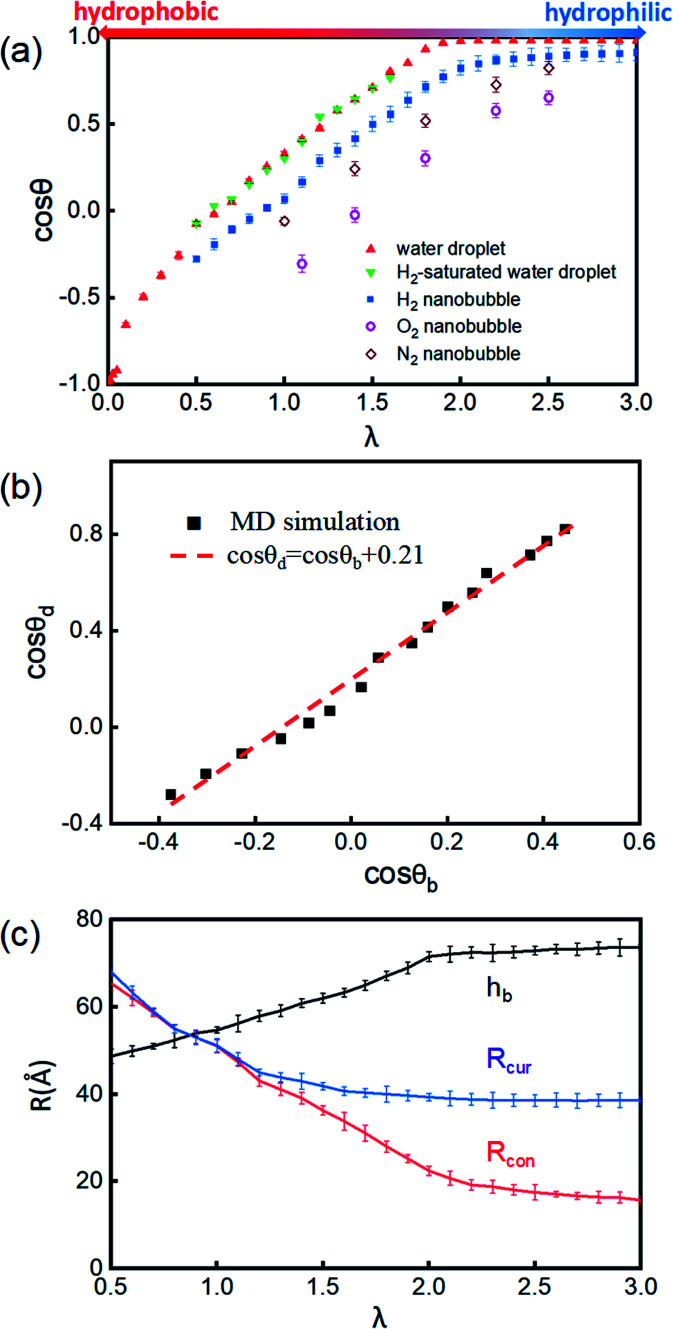
(a) Contact angles of the water droplets and INBs under different values of *λ*. The H_2_-INBs with *λ* < 0.5 will spread to cover the whole substrate, whose CA cannot be accurately obtained. (b) The linear relation between the CAs of H_2_-INBs and water droplets. Black squares denote the simulation results, and the red dashed line is fitted by least square multiplication with the linearly dependent coefficient of 0.9927. (c) The evolution of the contact radii (*R*_con_), the curvature radii (*R*_cur_), and the heights of H_2_-INB (*h*_b_) under various values of *λ*.

The cosine values of simulated CAs of H_2_/O_2_/N_2_-INBs, water droplets, and H_2_-saturated water droplets on substrates *versus λ* are demonstrated in Fig. 3a. All the CAs of the INBs (*θ*_b_) and the water droplet (*θ*_d_) have consistent trends with the surface energy parameter *λ*. However, when the surface gets more hydrophobic, O_2_-INB and N_2_-INB are more likely to form gas films like CO_2_-INB, so that the accurate value of bubble CA is difficult to obtain due to the limited size of the simulation box. Therefore, we take the H_2_-INBs (whose morphology is closer to the macrodroplet) as the example to comprehensively analyze the microscopic structure in detail. In the range of 0.5 < *λ* < 2.0, the values of cos *θ*_d_ and cos *θ*_b_ have a linear relation (Fig. 3b), which can be expressed by cos *θ*_d_ = cos *θ*_b_ + 0.21. Since it has been experimentally confirmed that the CA of water microdroplet is consistent with that of the macroscopic droplet (the same as macrobubble),^59^Fig. 3b is also provided with the relation between H_2_-INB and the macrobubbles. Besides the CA, the morphology of H_2_-INB can also be characterized by the radius of contact area (*R*_con_), the curvature radius of the spherical INB (*R*_cur_), and the bubble height (*h*_b_), which also show approximately linear relations with *λ* in the range of 0.5 < *λ* < 2.0 (Fig. 3c).

In the superhydrophobic region (*λ* < 0.5), the CA values of both nanobubble and nanodroplet approach to 180° (Fig. 3b). However, in the superhydrophilic region (*λ* ≥ 2.0), when the water droplet starts to completely wet the substrate (*θ*_d_ = *0*), the H_2_-INB still has a relatively large CA (*θ*_b_ = 34.8°). More interestingly, with the further increase of interfacial hydrophilicity, the CA of the INB tends to maintain a limit value (*θ*_b_ ≈ 25°) rather than approaching to zero. Correspondingly, *R*_con_ shows the same trend in this range (Fig. 3c). Combining with Fig. 2b and S3,† it is found that the nanobubble shape is no longer a regular spherical cap on a superhydrophilic surface; a neck-like structure with a height of ∼3 Å (one-molecule thick) appears at the triple line. In addition, such bubble necking structure is more obvious on the substrate with higher surface energy. It is known that the necking of a macrobubble usually appears during the pinch-off process, which can minimize the surface energy of the bubble.^66^ Here, the necking structure of H_2_-INB is due to the intrusion of water thin film with a minimum thickness (monolayer of water molecules) at the bubble/solid interface, implying the higher internal pressure of INB with a smaller curvature radius (due to the smaller CA on the liquid side) making it more difficult to deform on a superhydrophilic surface. Such phenomenon is also reflected from the context of INBs being extraordinarily stable and having extremely strong adhesion even on hydrophilic substrates. As shown in Fig. 3c, the unchanged curvature radius of bubbles in the region of the superhydrophilic surface also indicates that the necking structure can avoid the further increase of internal pressure of the nanobubble, thereby increasing the stability of the INB on a superhydrophilic surface. Therefore, unlike the traditional viewpoint that the superhydrophilic surface should be completely wet by water, the H_2_-INB can still stably sit on the superhydrophilic substrate, according to the present simulations. Finally, it is noteworthy that the CA of H_2_-saturated water droplet is identical to the CA of pure water droplet (Fig. 3a), indicating that for the H_2_-INB system, the surface tension change of water due to dissolved H_2_ is negligible in affecting the nanobubble behaviors. On the other hand, for the gases with higher solubility, such as CO_2_ and O_2_, their effects on surface tension may be more considerable, therefore affecting the morphology of the corresponding nanobubbles. Such point remains to be evidenced in future work.

### Microstructure of INBs

**Fig. 4:**
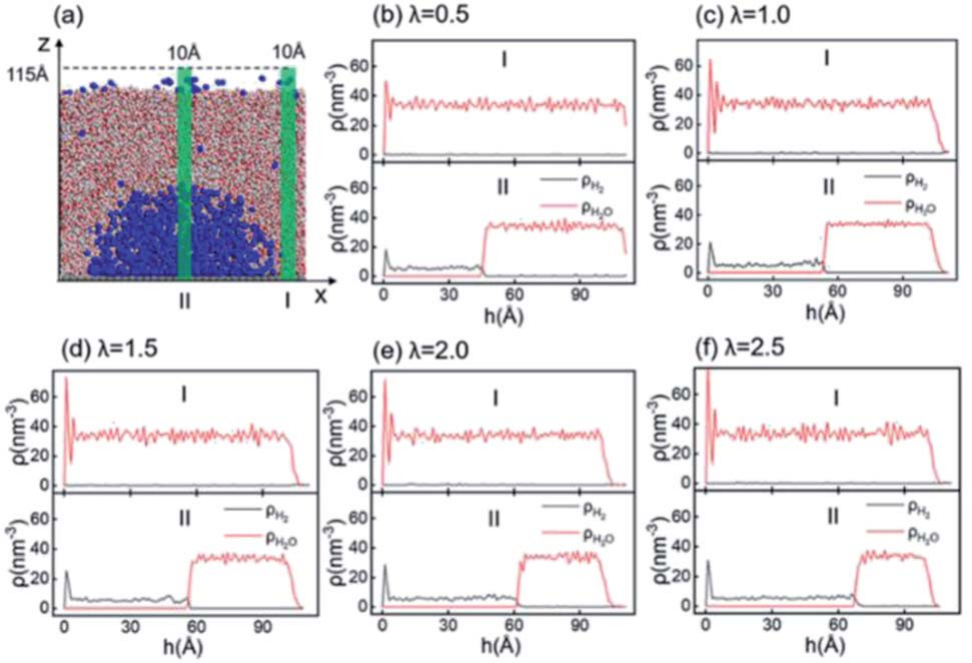
(a) Two 10 × 10 × 115 Å^3^ cuboid regions are taken for the density statistics. Region I nears the edge of the simulation box without contacting the nanobubble; region II is an area crossing through the mass center of the nanobubble. (b)–(f) Density distributions along the *z* direction inside region I and II under a series of *λ* values. The red curves represent the number density (*ρ*) of H_2_ molecules every cubic nanometer; black curve represents the number of water molecules in each cubic nanometer.

According to the Young–Laplace equation, the extremely high curvature of nanobubbles leads to much higher gas density inside the bubble, which has been already confirmed in many previous studies, and such high gas density has been considered to be related to the high stability of nanobubbles.^67–71^Fig. 4 shows the density distributions of H_2_ and H_2_O outside and inside the H_2_-INB covered regions (region I and region II in Fig. 4a) along the *z* direction (from the water–solid interface to the water–air interface) with different *λ* values. In region I (bulk water outside the bubble region), the number density of H_2_ molecules is close to zero due to the low solubility of H_2_ in water, and the average density of water of ∼33.6 H_2_O molecules per nm^3^ (∼1.005 g cm^−3^) is identical to the bulk water. The density of the main body of the nanobubble (region II) is ∼5.6 H_2_ atoms per nm^3^ (about 26.5% of liquid hydrogen density). Such high density of nanobubbles is also reported in previous investigations.^65^ In addition, there are two peaks significantly higher than the average value in the density profile of water, revealing the stratification of water close to the liquid/solid interface. A similar stratification is also observed for the density of H_2_ molecules in the vicinity of the solid/liquid interface, where a peak value is found at ∼3 Å high on the density profile, confirming the interfacial gas enrichment (IGE) phenomenon of nanobubbles discovered in previous works.^49^ Moreover, the maximum density of the gas layer (Fig. S4†) is found to be proportional to the interaction parameter *ε*_sg_/*ε*_ll_. Based on the IGE effect, Nikolai *et al.* proposed a bubble adsorption model, which indicates that the high-density pancake adsorption on the surface can reduce the surface energy, leading to the reduction of the gas–solid surface tension.^72–74^ It is noteworthy that the IGE behavior is not significant at the liquid–bubble interface, where the density of H_2_ is only slightly higher than in the bulk region of the bubble (Fig. 4b–f). Furthermore, the density distribution in region II also shows the fact that there are no H_2_O molecules inside the nanobubble. According to the density distribution (Fig. 4) and H_2_-INB snapshots on the hydrophilic surfaces (when *λ* > 2.0) (Fig. S3†), the INBs are composed of two parts: the bubble neck (which is about 2–3 gas molecule layers thick) and the main bubble, and the density of the bubble neck is much higher than that of the main bubble. Our simulations demonstrate that the internal structure of the INB is not homogenous, and with the increase of surface hydrophilicity, the bubble neck becomes more and more obvious. Therefore, we can consider that the density difference between the neck and the main body of the nanobubble leads to the higher stability and morphology change of the INB.

**Fig. 5:**
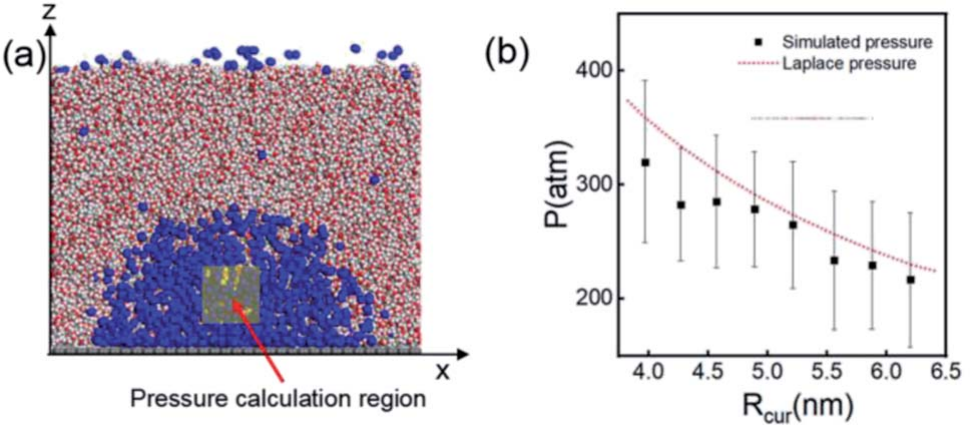
(a) A 20 × 20 × 20 Å^3^ volume is taken for pressure calculation, which is always inside the nanobubble. (b) Comparison of simulated internal pressure with the theoretical Laplace pressure. The time evolution of pressure when *R*_cur_ = 5.21 nm (*λ* = 1.0) is shown in Fig. S5.†

Another important and controversial feature of nanobubbles is the internal pressure. According to the traditional theory, the Laplace pressure of INBs is extremely large due to their ultra-small radius, which should be harmful to the stability of the bubble. Here, the internal pressure is predicted within the cubic region with the lattice of 2.0 nm (yellow area in Fig. 5a) after the bubble is fully relaxed by a ∼20 ns MD simulation. The pressure is computed based on the following formula:1
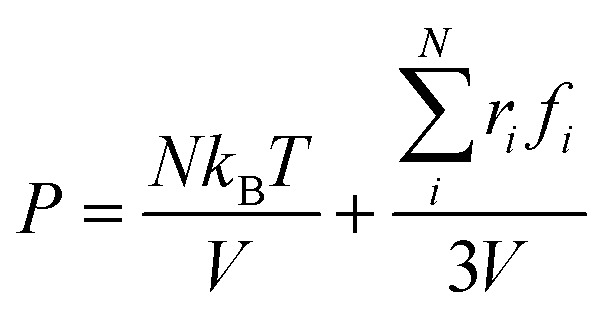
where *N*, *k*_B_, *T*, *V*, *r*_*i*_, and *f*_*i*_ denote the atomic number in the cubic region, Boltzmann constant, temperature, cubic volume, atomic position, and atomic force vector, respectively. As shown in Fig. 5b, the correlation between the calculated internal pressure and the curvature radius of INB is consistent with the trend of the pressure from the Laplace equation (proportional to 1/*R*_c_ with the assumption of constant surface tension), while the pressure values derived from the MD simulations are also slightly lower (∼12%) than the Laplace pressures, especially at the region of very small curvature radius. Our simulation results suggest that the Laplace equation is qualitatively correct for nanobubbles; however, the derivation of high-density gas from the ideal gas still reduces somewhat the internal pressure, which can help to stabilize the INB.^67^

### Size-dependence of INB morphology

**Fig. 6:**
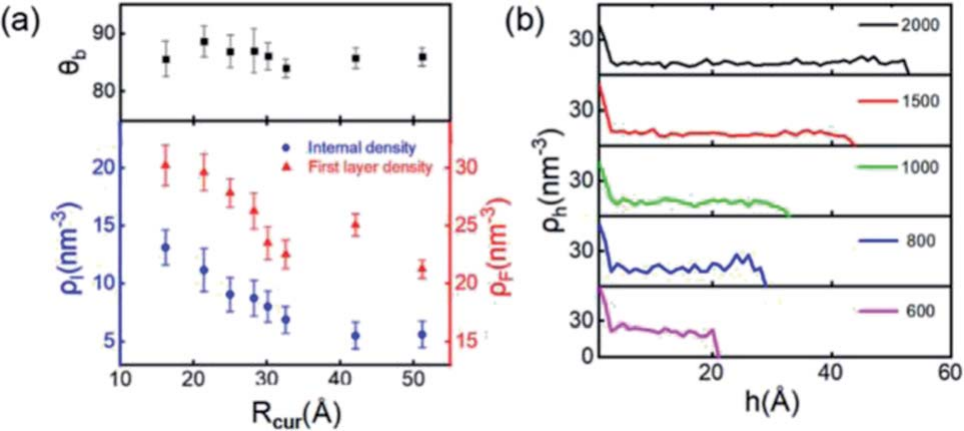
(a) The CAs and internal densities of the different-sized H_2_-INBs. The nanobubbles are initially formed with 500, 600, 700, 800, 900, 1000, 1500, and 2000 H_2_ molecules. *ρ*_h_ denotes the number of H_2_ molecules in 1 nm^3^. (b) The distributions of internal density along the *z* direction in the different sized H_2_-INBs.

Finally, we simulate the morphologies and behaviors of INBs containing 200, 500, 600, 700, 800, 900, 1000, 1500, and 2000 H_2_ molecules, corresponding to the *R*_c_ values in the range of 1–5 nm, to mimic the initial nucleation and growth processes, and investigate the size dependence of the INBs' CA. The systems are set to make sure that all the initial numbers of H_2_ molecules placed inside the bubble meet the condition of system saturation due to the extremely low solubility of hydrogen in water. It is found that the cluster with 200 H_2_ molecules cannot appear as an INB, while all the other clusters can form stable INBs with unchanged CAs during MD simulations (simulation time > 10 ns). As shown in Fig. 6a, the CAs of H_2_-INBs with *R*_c_ values ranging from 1.5 to 5 nm show almost identical CAs (*θ* ≈ 86°). By contrast, the gas density inside the INB and the first H_2_ layer density have an apparently negative correlation with the bubble radius (red curve in Fig. 6a). Furthermore, the hydrogen gas is almost uniformly distributed inside the INBs with similar IGE peaks at liquid–air interfaces, as shown in Fig. 6b.

Previous studies have already gained a physical picture of the formation process of INBs with pinning sites: gas molecules first blanket the pinning surface to form a pancake, and then transform into an increasingly full spherical coronal nanobubble without changing the three-phase contact area.^15,16^ Our simulations further reveal the mechanism of nucleation of INBs on the atomic flat surface without any pinning points, where the growth of INB follows a constant contact angle (CCA) mode. In addition, gas density is also an important indicator for nanobubble nucleation. The present simulations show that the critical gas nuclei may adopt a very high density (>60% of the density of liquid hydrogen) in the initial nucleation stage, then the density rapidly decreases to ∼25% of the liquid–H_2_ density during the growth process.

### Modified Young's equation for INBs

It is well known that the CA of a droplet on a smooth surface can be described by Young's equation,2
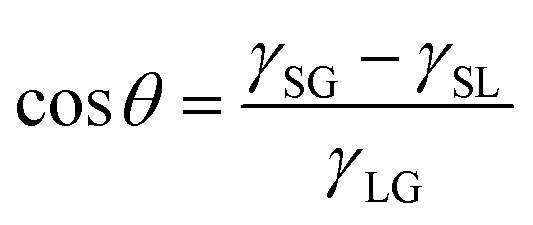
where *γ*_SG_, *γ*_SL_ and *γ*_LG_ respectively denote the solid–vapor, solid–liquid, and liquid–vapor interfacial energies. Under ambient conditions, *γ*_LG_ is synonymous with the liquid surface tension. When the surface energy of substrate varies within a certain range, we can consider that the interfacial structure does not apparently change. As a result, the values of both *γ*_SG_ and *γ*_SL_ are proportional to the LJ-potential coefficient *λ* of the substrate, leading to the linearity between cos *θ* and *λ* (*λ* = 0.5–2.0 in the present work), as displayed in Fig. 3a. Due to the higher gas density in the nanobubble, the CA of INB can be described by a modified Young's equation,3
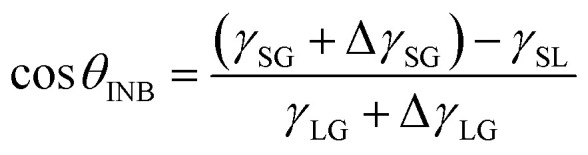
where Δ*γ*_SG_ denotes the increase of interfacial energy caused by the solid–vapor IGE behavior, and Δ*γ*_LG_ represents the increased interfacial energy due to the increased gas density in the INB. The solid surface and gas molecule have attractive interaction with a negative value of Δ*γ*_SG_, and the IGE is not obvious for the liquid–vapor interface, indicating the value of Δ*γ*_LG_ is mainly relevant to the inner gas density of INBs. As shown in Fig. 4b–f, the density distributions indicate the same IGE structures in INBs on surfaces with various wettabilities. Therefore, similar to the case of droplet wetting, the values of *γ*_SG_, Δ*γ*_SG_, and *γ*_SL_ increase linearly with *λ*, leading to a linear relation between cos *θ*_INB_ and *λ* in a certain range (0.5< *λ* < 2.0) as well, as shown in Fig. 3a. And eqn (3) is also helpful to understand the CAs of INBs with different gas species. Since the H_2_ molecule has the weakest interaction with the substrate, leading to smaller Δ*γ*_SG_ than other gas species, the CA of H_2_-INB is closest to that of a water droplet. In contrast, the large value of Δ*γ*_SG_ for CO_2_ makes the right side of eqn (3) lower than −1, leading to a super large CA of CO_2_-INB of ∼180°. Finally, in the size-dependent study, the IGE peak area of INBs almost remains constant at different bubble sizes (Fig. 6b), indicating the Δ*γ*_SG_ also remains constant at different sizes. Due to the much weaker H_2_–water and H_2_–H_2_ interactions (pure van der Waals interaction) than the water–water hydrogen bonding interaction, the denominator of eqn (3) is mainly contributed by *γ*_LG_, which can also be considered as a constant. Thereby, the CA of INBs does not demonstrate obvious size dependence in the size range of the present simulations (1.5 nm < *R*_c_ < 5.0 nm).

## Conclusions

In summary, MD simulations have been carried out to comprehensively investigate the main factors, including the gas species, surface energy of substrate, and bubble size, that affect the morphology and behavior of INBs. It is found that H_2_ can form an INB on an atomic flat surface with a shape of a macrodroplet; O_2_ and N_2_ can also form stable INBs but with significantly larger CAs than that of H_2_-INB, while CO_2_ is more likely to form a gas film (pancake) structure on the surface due to its strong gas–solid interaction. The CAs of INBs show a linear relation with the strength of van der Waals interaction of the substrate, demonstrating a consistent trend with the CA of water droplets. The high density and high pressure of the gas inside the INB are also confirmed, as well as the strong IGE effect at the solid–liquid interface. In addition, it is also found that the density of gas is sensitive to the bubble size at the initial stage of bubble nucleation, while the bubble CA remains almost constant. It is further revealed that all the above simulation results can be understood by a modified Young's equation. Our simulations give deep insights into the morphology and microstructure of nanobubbles, which are also of great importance to the relevant applications.

## Conflicts of interest

There are no conflicts to declare.

## Supplementary Material

NA-004-D2NA00009A-s001
