# Supplementary material for: Unraveling the effects of gas species and surface wettability on the morphology of interfacial nanobubbles
Source: Nanoscale Adv. 2022 May 24;4(13):2893–901. doi: 10.1039/d2na00009a (PMC9418701; doi:10.1039/d2na00009a)
Supplement: NA-004-D2NA00009A-s001 [file NA-004-D2NA00009A-s001.pdf]

Electronic Supplementary Material (ESI) for Nanoscale Advances.
This journal is © The Royal Society of Chemistry 2022

Supplementary Information

Unraveling the Effects of Gas Species and Surface Wettability on Morphology of Interfacial Nanobubbles

Kadi Hu[a], Liang Luo[b], Xiaoming Sun[a,b], and Hui Li*[a]

a. Beijing Advanced Innovation Center for Soft Matter Science and Engineering, Beijing University of Chemistry Technology, Beijing 100029, PR China.

b. State Key Laboratory of Chemical Resource Engineering, Beijing University of Chemical Technology Beijing 100029, PR China.

*E-mail: hli@mail.buct.edu.cn

Contents

Supporting Figures……………………………………………………………………………………………S1

Supporting Tables………………………………………………………………………………………………S6

Part I Supporting Figures

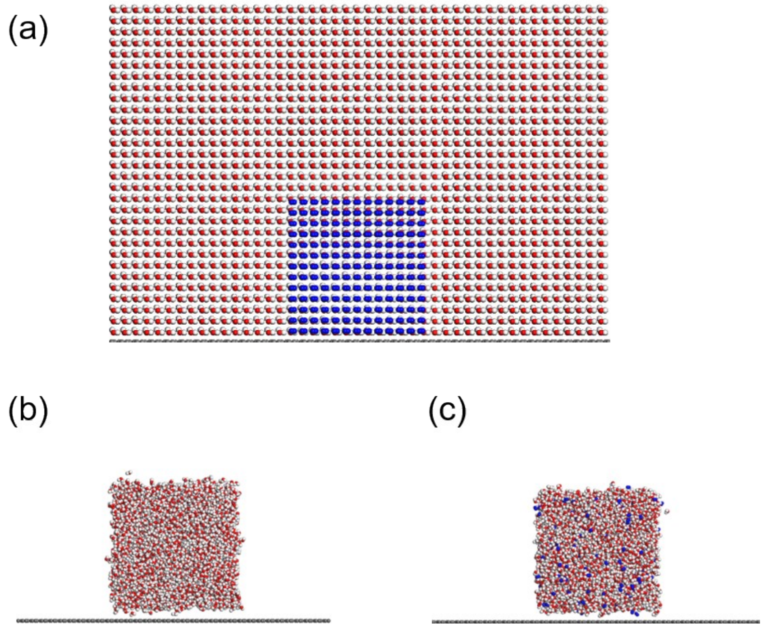

Fig. S1 (a) Initial structure of the H_2 nanobubble system, which contains 57375 H_2O molecules, 2197 H_2 molecules, and 7524 C atoms. (b) Initial state of the pure water droplet, which contains 5000 H_2O molecules. (c) Initial state of the water droplet mixed with H_2, which contains 5000 H_2O molecules and 150 H_2 molecules.

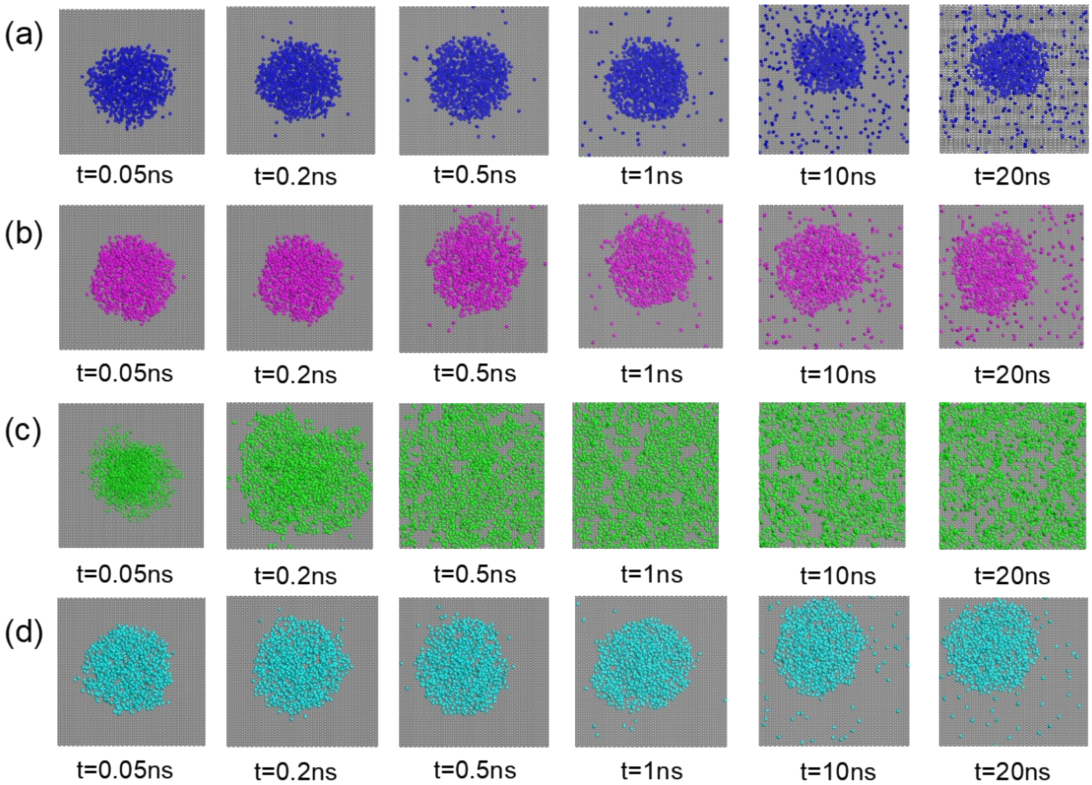

Fig. S2 Time evolution of the (a) H_2, (b) O_2, (c) CO_2, and (d) N_2 nanocluster morphologies on atomistic flat surfaces (plan views).

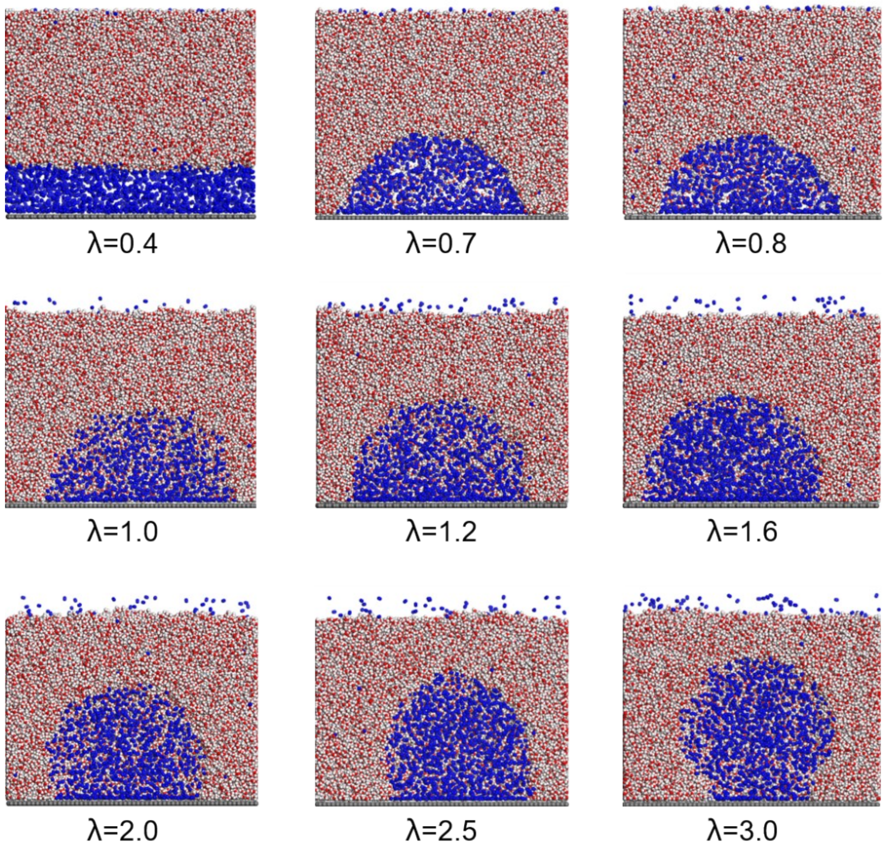

Fig. S3 Snapshots of the nanobubbles on surfaces (side views) with different values of λ, which from small to large denotes the wettability changing from hydrophobic to hydrophilic.

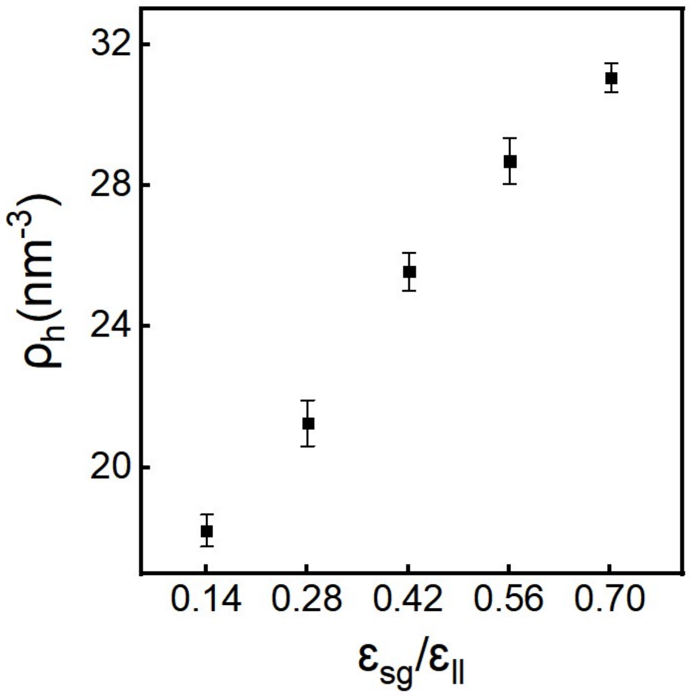

Fig. S4 Relationship between the density of first H_2 layer and the interaction parameter $\varepsilon_{sg}/\varepsilon_{ll}$. Each $\varepsilon_{sg}/\varepsilon_{ll}$ is corresponding to the surfaces of different wettability from λ=0.5 to λ=2.5.

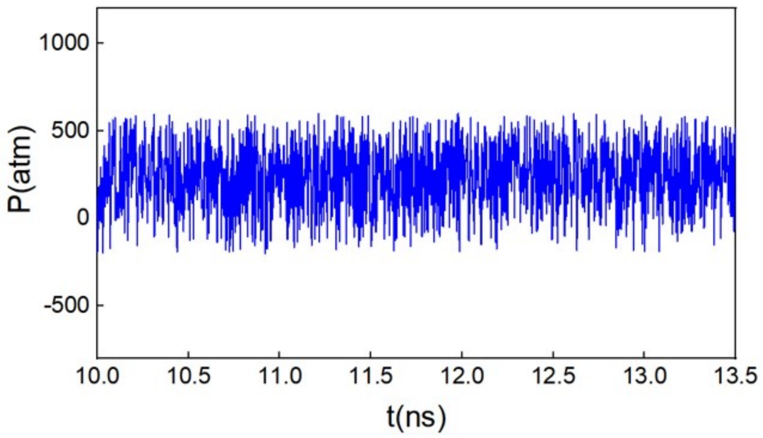

Fig. S5 Time evolution of the internal pressure of surface nanobubble when $R_{cur} = 5.21$ nm (λ=1.0). The pressure fluctuates a lot as the simulation goes.

Part II Supporting tables

Table S1 Interaction parameters between atoms.[1] C_G: the atoms of graphene; O_W: the O atom of H_2O; H_W: the H atoms of H_2O; H_H: the atoms of H_2; O_O: the atoms of O_2; O_C: O atom of CO_2; C_C: C atom of CO_2. N: N atom of N_2.

	C_G	O_W	H_W	H_H	O_O	O_C	C_C	N
$\mathcal{E}$(kcal/mol)	0.086	0.155	0	0.022	0.103	0.103	0.183	0.074
σ (Å)	3.400	3.169	0	2.683	3.006	3.006	2.937	3.292
Charge(e)	0	-0.8476	0.4238	0	0	-0.3256	0.6512	0

The water molecules are treated by the SPC/E model. The Lennard-Jones potential ($E = 4\varepsilon[\left(\frac{\sigma}{r}\right)^{12} - \left(\frac{\sigma}{r}\right)^{6}]$) with a cutoff of 12.0 Å is employed for the Van der Waals interaction. The interaction parameters between different types of atoms are calculated through the Lorentz-Berthelot combining rules ($\varepsilon_{ij} = \sqrt{\varepsilon_{ii}\varepsilon_{jj}}$, $\sigma_{ij} = \frac{\sigma_{ii+} + \sigma_{jj}}{2}$).

References

1 J. P. Bouanich, Site-site Lennard-Jones Potential Parameters for N_2, O_2, H_2, CO and CO_2, *J.*

Quant. Spectrosc. RA., 1992, **47**,243-250.
